# Effect of the lipoxygenase inhibitor baicalein on bone tissue and bone healing in ovariectomized rats

**DOI:** 10.1186/s12986-018-0327-2

**Published:** 2019-01-11

**Authors:** Dominik Saul, Marie Weber, Marc Hendrik Zimmermann, Robyn Laura Kosinsky, Daniel Bernd Hoffmann, Björn Menger, Stefan Taudien, Wolfgang Lehmann, Marina Komrakova, Stephan Sehmisch

**Affiliations:** 1 Department of Trauma Surgery, Orthopaedics and Plastic Surgery, University Medical Center Goettingen, 37075 Goettingen, Germany; 2 Department of General, Visceral and Pediatric Surgery, University Medical Center Goettingen, 37075 Goettingen, Germany; 3 Division of Infection Control and Infectious Diseases, University Medical Center Goettingen, 37075 Goettingen, Germany

**Keywords:** Baicalein, Lipoxygenase inhibitor, Fracture healing, Osteoporosis, Biomechanics

## Abstract

**Background:**

Osteoporosis is one of the world’s major medical burdens in the twenty-first century. Pharmaceutical intervention currently focusses on decelerating bone loss, but phytochemicals such as baicalein, which is a lipoxygenase inhibitor, may rescue bone loss. Studies evaluating the effect of baicalein in vivo are rare.

**Methods:**

We administered baicalein to sixty-one three-month-old female Sprague-Dawley rats. They were divided into five groups, four of which were ovariectomized (OVX) and one non-ovariectomized (NON-OVX). Eight weeks after ovariectomy, bilateral tibial osteotomy with plate osteosynthesis was performed and bone formation quantified. Baicalein was administered subcutaneously using three doses (C1: 1 mg/kg BW; C2: 10 mg/kg BW; and C3: 100 mg/kg BW) eight weeks after ovariectomy for four weeks. Finally, femora and tibiae were collected. Biomechanical tests, micro-CT, ashing, histological and gene expression analyses were performed.

**Results:**

Biomechanical properties were unchanged in tibiae and reduced in femora. In tibiae, C1 treatment enhanced callus density and cortical width and decreased callus area. In the C3 group, callus formation was reduced during the first 3 weeks after osteotomy, correlating to a higher mRNA expression of *Osteocalcin*, *Tartrate-resistant acid phosphatase* and *Rankl*. In femora, baicalein treatments did not alter bone parameters.

**Conclusions:**

Baicalein enhanced callus density and cortical width but impaired early callus formation in tibiae. In femora, it diminished the biomechanical properties and calcium-to-phosphate ratio. Thus, it is not advisable to apply baicalein to treat early bone fractures. To determine the exact effects on bone healing, further studies in which baicalein treatments are started at different stages of healing are needed.

**Electronic supplementary material:**

The online version of this article (10.1186/s12986-018-0327-2) contains supplementary material, which is available to authorized users.

## Background

With a prevalence of approximately 10.3% in Germany and 8.9 million fractures annually worldwide, osteoporosis is a common and relevant medical burden [1, 2]. One of the major osteoporotic fractures after those of vertebrae is in the proximal femur [3, 4], while the osteoporotic tibial fractures are frequently missed [5, 6]. Modern therapy consists of calcium and vitamin D supplementation as well as anabolic and anti-catabolic treatments [7, 8] because of age-related deficiencies. Recent promising therapeutic approaches include lipoxygenase (LOX) inhibitors, which were found to ameliorate bone density [9]. The subgroup of 5-lipoxygenase (5-LOX) inhibitors were found to inhibit osteoclast formation [10] and enhance bone formation [11]. One of these lipoxygenase inhibitors is baicalein, a phytochemical agent extracted from the plant *Scutellaria baicalensis* Georg. It appears to inhibit both 12-LOX and 15-LOX, leading to an antioxidative effect. Baicalein also inhibits 5-lipoxygenase by preventing translocation of 5-LOX to the nuclear envelope and p38 phosphorylation [12].

Further studies have revealed inhibition of the NF-κB signalling by baicalein. Through this pathway, baicalein suppresses the function of TNFα, IL-6 and IL-1β, which are mediators of inflammation cascades [13]. Additionally, baicalein was reported to inhibit receptor activator of NF-κB ligand (RANKL) and, contrarily, induce tartrate-resistant acid phosphatase (TRAP)-activity and thereby has an impact on osteoclastogenesis and bone resorption in bone erosive diseases in general [14] and rheumatoid arthritis in particular [15, 16]. More generally, the bone resorptive activity of osteoclasts was inhibited by baicalein by inhibiting osteoclast differentiation and promoting osteoclast apoptosis [17]. In addition to these primary inflammation-based activities, baicalein was found to activate alkaline phosphatase by the mammalian target of rapamycin complex 1 (mTORC1) pathway and thus induce osteoblast differentiation markers and increase distinct bone parameters in the distal femur [18].

Hence, anticipating an effect on bone, we recently demonstrated that baicalein improved cortical bone but not trabecular parameters in lumbar vertebrae of ovariectomy-induced osteopenic rats [19]. Notably, as described in our previous studies, the anti-inflammatory effect was favourable regarding the muscular structure [20]. Encouraged by these findings in vertebrae and muscle, we decided to investigate the effect of baicalein on the fracture healing and bone structure of long bones such as the tibia and femur in an osteoporotic rat model.

## Methods

### Animals and treatment

Sixty-one female, three-month-old Sprague-Dawley rats (Winkelmann, Borchen, Germany) were kept at 20 °C and a relative humidity of 55% in Makrolon IV® cages. These rats were the same as reported in other studies investigating muscle and spine properties and serve as a model of osteoporosis [19–21]. After one acclimatization week, experiments were conducted in accord with the ethical standards of animal care (application number G14/1530).

The 13-week-old rats underwent bilateral ovariectomy (OVX) or Sham-surgery (NON-OVX), as previously described [22]. The former group served as osteoporotic control group, the latter group as intact healthy controls. All surgical procedures were carried out under ketamine/Domitor anaesthesia (0.1 ml/100 g BW i.p.). After shaving, anaesthesia and disinfection, the skin was incised left and right of the abdomen. In the next step, ovaries were dissected, clamped and removed before the wound was closed. No analgesia was needed after surgery.

Eight weeks later, when OVX rats developed osteoporotic changes in bone [18], bilateral osteotomy of the tibial metaphysis with plate osteosynthesis was performed. Osteotomy was performed 7 mm distal to the knee surface with a pulsed ultrasound saw as described previously [23, 24]. Osteotomy gap of 0.5 mm was created, and a five-hole T-shaped titanium fixation plate (57–05140, Stryker Trauma, Selzach, Switzerland) was fixed with the aid of four 1.2-mm screws to the anterior-medial surface. Based on previous studies [25, 26], we assumed that at around this time point, i.e., eight weeks after OVX, the rats would have developed osteoporosis. Baicalein treatments were started on the next day after osteotomy. Baicalein (98%, Sigma-Aldrich Chemie GmbH, Munich) was dissolved in pure dimethyl sulfoxide (DMSO), while both control groups (NON-OVX and OVX, *n* = 10) received DMSO alone. Baicalein was injected subcutaneously at different doses, as previously described [27, 28] (C1: 1 mg/kg BW; C2: 10 mg/kg BW; and C3: 100 mg/kg BW) in three groups with ten to thirteen animals each. The injections were performed every 24 h for four weeks.

**Fig. 1:**
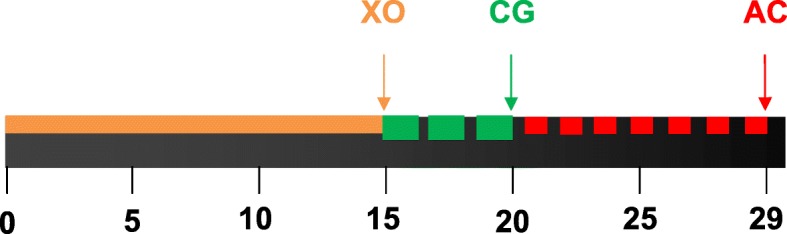
Schematic flowchart of the application time of fluorochromes. XO was injected on day 15 and stained the callus built up to day 15. CG was applied on day 20 and stained the osseous callus formed between days 15 and 20. AC was applied on day 29 after osteotomy and labelled the callus built between days 20 and 29

To analyse the dynamics of callus formation, the newly synthesized bone was labelled with fluorescent dyes according to van Gaalen et al. [29]. In this rat model, 28 days’ follow-up is the ideal condition for fracture healing and maximum callus reaction [30]. Xylenol orange (XO, 90 mg/kg BW), calcein green (CG, 10 mg/kg BW) or alizarin complexone (AC, 30 mg/kg BW) was injected subcutaneously on the fifteenth (XO), twentieth (CG) or twenty-ninth (AC) days after osteotomy (Fig. 1).

Twenty-nine days after osteotomy, the anaesthetized rats were decapitated, blood samples were collected, and sera were stored at − 20 °C. Uteri were extracted and weighed, femora and tibia were dissected free of soft tissues, and the tibia osteosynthesis material (plate and screws) was removed. Left or right femora chosen randomly were stored at − 20 °C for biomechanical, micro-computed tomographical (micro-CT) and ashing analyses. Right or left tibiae chosen randomly were stored at − 20 °C for micro-CT, biomechanical and histological analyses.

Metaphyseal clips of contralateral tibia that contained a callus and did not include the epiphysis or growth plate were immersed in liquid nitrogen immediately after extraction for up to 5 h. For further storage, the samples were transferred to the freezer and stored at − 80 °C until gene expression analysis.

### Three-point bending test of tibia callus and femur

Biomechanical properties of femora and tibia were tested with a three-point bending test as previously described [31–33] using a Zwick machine (145,660 Z020/TND; Ulm, Germany, Additional file 1: Figure S1A-E). Bones were positioned at the base of the Zwick machine. After that, a stamper was depressed onto the long bone at 50 mm/min and an initial force of 1 N to fix the bone onto the plate. Tibia was placed on the aluminium base and loaded 2–3 mm distally to the osteotomy at the tuberositas tibiae [32] to assess biomechanical properties of the callus. In femora, the medial and lateral condyle were removed. The femoral head was placed on the base and in a 4-mm deepening on the end of the plate and then loaded to the trochanteric region [31].

Testing showed a linear increase and was finalized when the curve declined before the tibia callus was broken. The femur was tested until a fracture in the trochanteric region occurred.

Recording was performed using the testXpert software (Zwick GmbH & Co. KG, Ulm, Germany). We quantified the maximum load (Fmax) and stiffness as described by Tezval et al. [34, 35] and Stuermer et al. [33]. The Fmax is the highest force that the bone can withstand, while stiffness is the slope of the linear increment of the curve.

### Micro-CT analysis

For analyses of bone volume and density, femora and tibiae were scanned with a Quantum FX micro-CT device (Caliper Sciences, Hopkinton, MA, USA) at 70 kVp and 200 μA with a 30 Hz detector frame rate, 360° rotation, and 3600 projections, resulting in a 40 × 40 × 40 μm^3^ effective voxel size. Fixed filters of 0.5 mm aluminium and 0.06 mm copper were used to remove low-energy X-rays; a noise filter was not used.

A program that was developed in our laboratory (3D OsteoAnalyze) was used to measure bone parameters according to ASBMR criteria [36, 37]. A later version, Scry v6.0 software (Kuchel & Sautter UG, Bad Teinach-Zavelstein, Germany), is commercially available [38]. Standard thresholds for callus or trabecular bone (from 0.5 to 1.1 g/cm^3^) and cortical bone (from 1.1 g/cm^3^ to the highest measured threshold), soft tissue (from − 0.1 to 0.5 g/cm^3^) and total tissue (from − 0.1 g/cm^3^ to the highest threshold) were calculated by taking the mean of 15 measurements of visually detected thresholds (three samples from each of the five groups) and used for all samples [18]. A phantom block with five hydroxyapatites of defined mineral densities (0.2, 0.4, 0.6, 0.8 and 1.0 g/cm^3^) was scanned with each bone to convert the data into the bone mineral density.

The analysis of tibiae focused on the area from 2.5 mm proximally and distally to the osteotomy site as described by Komrakova et al. [24]. Callus, cortical and total bone densities (g/cm^3^), callus and total volume (as amount of soft tissue volume, callus volume and cortical volume in mm^3^) and bone volume fraction (BV/TV) as well as soft tissue volume were assessed.

In femora, the femoral head was the region of interest. The femoral head was cut in the transition zone from the collum femoris to the trochanter major. By defined thresholds, density and volume could be calculated with the 3D OsteoAnalyze software. Measured parameters were BV/TV, total BMD, cortical BMD, trabecular BMD and total and soft tissue volumes.

### Histological analyses

For callus analyses, tibiae were deposited in ascending ethanol concentrations and finally fixed in methyl methacrylate (Merck, Darmstadt, Germany). Next, 150-μm sections were cut parallel to the long axis of the bone and at a 90° angle to the implant bed with a diamond saw microtome (Leica SP 1600, Leica Instruments GmbH, Nussloch, Germany) [39]. Three central sections were microradiographed with a Faxitron Cabinet X-ray system (Hewlett-Packard, Buffalo Grove, IL) and Kodak Industrex film (SR45, 100 NIF; Kodak, Paris, France). The sections and microradiographs were digitalized with a Leica DC300F camera and a zoom stereoscope (Leica MZ75). Using the MetaMorph Basic Acquisition 185 Software (Leica Mikrosysteme Vertrieb GmbH, Wetzlar, Germany), the area of interest was 2.5 mm proximal and distal from the osteotomy site with ventral, dorsal and endosteal tibia aspects. Measured parameters of the histological sections were as follows: callus area labelled with different fluorochromes, where an XO-labelled callus was formed between days 0 and 15; CG-callus between days 16 and 20; and AC-callus between days 21 and 29 (Fig. 1). Since the XO area was too small, it was measured along with the CG area, i.e., XO + CG was the area of bone that was labelled by both XO and CG fluorochromes. Total callus area was calculated as a sum of mineralized fluorochrome labelled callus areas. Further callus area fraction was calculated by the software as the ratio of fluorochrome-labelled callus area to the total callus area, which included the black background of the images according to the tibia aspects (ventral, endosteal and dorsal).

The time point of the first bridging by a callus was detected microscopically (Leica microscope, Leitz DM RXE) by analysis of at least ten sections of each tibia [40]. Each histological section was analysed on the occurrence of the first bridging. For example, if the bridging appeared as a red thin thread, the day of bridging was shortly before the injection (AC, day 28), whereas a thicker red band indicated the bridging on day 26 (Fig. 5). The analysis was performed by the same person blinded to the treatments. After analysis of all sections, the first bridging day was taken for each tibia and further evaluated (Table 1).

**Table 1:** Day of first osseous callus bridging occurring in NON-OVX rats or OVX rats treated with different concentrations of baicalein

	NON-OVX	OVX	Baicalein C1	Baicalein C2	Baicalein C3
Day of bridging	28	26	29	28	26
	20	29	28	27	28
	10	29	19	25	19
	26	27	23	20	26
	21			28	
	20			28	
Mean ± SD	21 ± 6	28 ± 2	25 ± 5	26 ± 3	25 ± 4
Tibia bridging (n, % of total n)	6 (67%)	4 (44%)	4 (57%)	6 (86%)	4 (40%)
Total (*n)*	9	9	7	7	10

Using microradiographs, callus area fraction (ventral, dorsal, endosteal), callus width (ventral, dorsal) and cortical width (ventral and dorsal distal to the osteotomy line) were measured according to [32, 41]. Callus area fraction was depicted as a percentage of calcified callus area to the non-calcified callus area within the 3 tibia aspects [39, 40] or the percentage of white calcified tissue to the black background on the microradiographs. All these measurements have been previously described in detail [25, 39, 42].

### Ashing of the femur

To determine organic and anorganic contents of the femoral body, femora were ashed at 750 °C for one hour. The anorganic mass and organic mass were calculated relative to the wet mass of respective femur recorded prior to ashing. The calcium content was quantified with an atomic absorption spectrometer (4100; PerkinElmer, Baesweiler, Germany), and orthophosphate content was scaled by the colourimetric method (Spectral Photometer DM4; Zeiss, Jena, Germany) [43].

### Gene expression analysis of the tibia at the osteotomy site

RNA was extracted as previously described [44]. Tibial calluses were homogenized, and RNA was isolated using the RNeasy™ Mini Kit (Qiagen, Hilden, Germany). Reverse transcription followed using Superscript™ RNase H (Promega, Mannheim, Germany). Quantitative real-time PCR (qRT-PRC) was used to determine the expression of the rat genes arachidonate 5-lipoxygenase (*5-lox*), arachidonate 12-lipoxygenase (*12-lox*), arachidonate 15-lipoxygenase (*15-lox*), alkaline phosphatase (*Ap*), insulin-like growth factor 1 (*Igf-1*), interleukin 6 (*Il-6*), interleukin 7 (*Il-7*), bone gamma-carboxyglutamate protein (Osteocalcin, *Oc*), receptor activator of nuclear factor kappa B ligand (TNF superfamily member 11, *Rankl*), transforming growth factor alpha (*Tgf-α*), and tartrate-resistant acid phosphatase (*Trap*). The PCR was based on SYBR Green detection with an iCycler (CFX96, Bio-Rad Laboratories, Munich, Germany). Ready-to-use primers for the measurement of mRNA expression were synthesized by Qiagen (QuantiTect Primer Assays, Hilden, Germany). The expression levels were normalized to beta-2 microglobulin and calculated using the 2^-ΔΔCT^ method [45].

### Statistics

Statistical analyses were conducted using GraphPad Prism 5.04 (GraphPad Software, San Diego, CA, USA). One-way analysis of variance (ANOVA, *p* < 0.05) was applied to reveal the impact of the treatments on variables. Differences between individual means were estimated using Tukey’s post hoc test for multiple comparisons with a significance level of 0.05 (95% confidence interval) (***p ≤ 0.001, **p ≤ 0.01, *p ≤ 0.05.). Data in the figures are shown as the means and standard errors of the means. The Grubbs test was performed for outliers, but none were detected.

## Results

### Rat characteristics

Prior to the experimental procedures, BW did not differ among the groups, while at the end, the OVX group was significantly heavier than the NON-OVX group (published by our group in [19, 20]). Baicalein did not alter the BW. The weight of uteri was significantly lower in all OVX groups irrespective of baicalein treatment (Additional file 2: Figure S2D). Weight of femora did not differ among the groups at the end of the treatments (Additional file 2: Figure S2C).

### Three-point bending test of tibia callus and femur

**Fig. 2:**
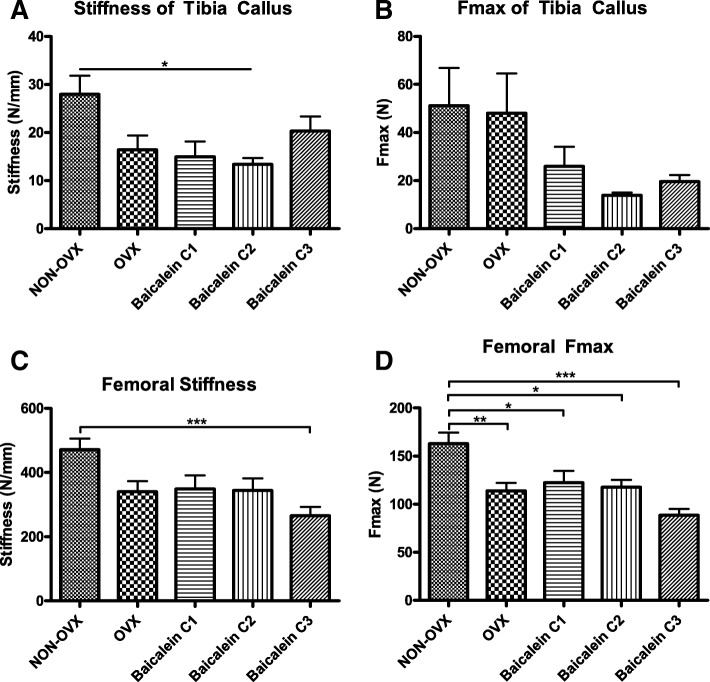
Bending tests of the tibia at the osteotomy site and femora at the trochanteric region. Stiffness (**a** and **c**), maximal force (Fmax, **b** and **d**). Stiffness was affected by baicalein (C3) in femur and by C2 in tibial callus. Ovariectomy diminished the Fmax in femora, whereas in tibiae no differences were detected in Fmax due to the high variation between the groups. ***p ≤ 0.001, ** ≤ 0.01, *p ≤ 0.05 (for tibia callus (**a** and **b**) NON-OVX *n* = 6, OVX *n* = 8, C1 *n* = 7, C2 *n* = 7, C3 *n* = 7; for femur (**c** and **d**): NON-OVX *n* = 10, OVX *n* = 9, C1 *n* = 10, C2 *n* = 9, C3 *n* = 10)

Bone biomechanical properties were quantified with the Zwick device. In tibia callus, stiffness was slightly impaired in the C2 group compared to NON-OVX (Fig. 2a). In Fmax, no statistically significant differences were detected (Fig. 2b). None of the baicalein-treated groups showed significant differences compared to the OVX group. In femora, there was reduced stiffness in C3 baicalein-treated group compared to the NON-OVX group (Fig. 2c). A similar trend was indicated for Fmax as well: Maximum force (Fmax) was significantly reduced in femora in all OVX groups, whether treated with baicalein or not, compared to the NON-OVX group (Fig. 2d).

### Micro-CT

#### Tibia

**Fig. 3:**
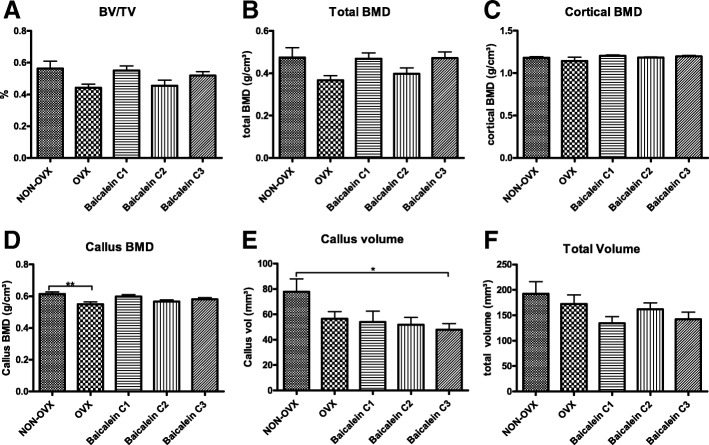
Micro-CT of osteotomy site in tibia revealed only minor effects of baicalein. The parameters measured in tibiae were bone volume/total volume (BV/TV, **a**), total BMD (**b**), cortical BMD (**c**), and callus BMD (**d**), as well as callus volume (**e**) and total volume (**f**). For callus BMD (**d**), ovariectomy led to reduced values, while callus volume (**e**) was significantly reduced at the highest baicalein concentration. ***p ≤ 0.001, **p ≤ 0.01, *p ≤ 0.05 (NON-OVX *n* = 9, OVX *n* = 9, C1 *n* = 9, C2 *n* = 9, C3 *n* = 10)

Next, micro-CT was performed to assess bone volume/total volume (BV/TV) as well as BMD. In tibiae, BV/TV (Fig. 3a), total BMD (Fig. 3b), cortical BMD (Fig. 3c) and total volume (Fig. 3f) were not different between the groups. Callus BMD was significantly reduced in the OVX group compared to the NON-OVX group (Fig. 3d), while all baicalein treatments caused no measurable effect. Callus volume, on the other hand, was highest in the NON-OVX group, though it was only significantly higher than the highest baicalein concentration group (Fig. 3e).

#### Femur

**Fig. 4:**
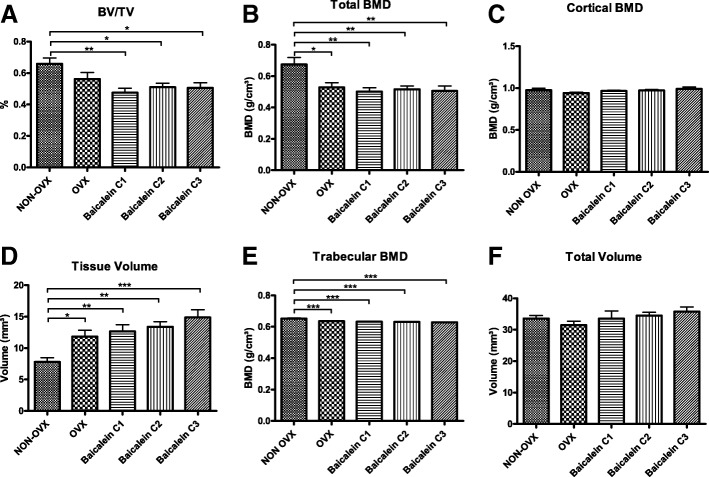
Micro-CT of femoral head showed no effect of baicalein. For BV/TV (**a**), total BMD (**b**) and trabecular BMD (**e**), OVX led to reduced values, which could not be rescued by baicalein. No differences in cortical BMD (**c**) or in total volume (**f**) could be measured. For tissue volume (**d**), OVX increased values significantly, with an increasing effect of baicalein. BV/TV was significantly diminished in all baicalein groups ***p ≤ 0.001, **p ≤ 0.01, *p ≤ 0.05 (NON-OVX *n* = 10, OVX *n* = 9, C1 *n* = 10, C2 *n* = 9, C3 *n* = 10)

The ratio of bone volume to total volume revealed reduced values for all baicalein-treated groups (Fig. 4a). Total BMD (Fig. 4b) and trabecular BMD (Fig. 4e) were lower in all OVX groups compared to the NON-OVX group. No differences in cortical BMD (Fig. 4c) or in total volume (Fig. 4f) were observed, whereas the tissue volume was higher in all OVX groups (Fig. 4d).

### Histological analyses of fluorescence-labelled sections of tibia

**Fig. 5:**
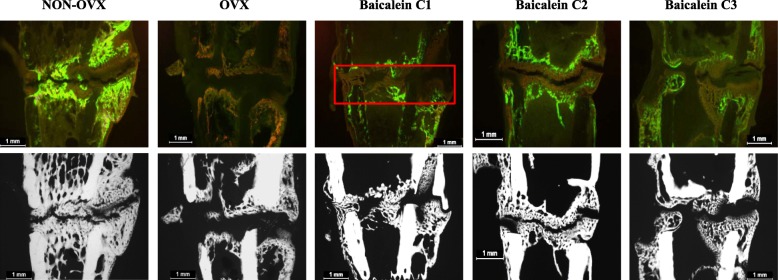
Fluorochrome-labelled sections and microradiographs of the metaphysis of the tibiae. Fluorochrome-labelled sections (first row) and microradiographs (second row) in the NON-OVX (**a**), OVX (**b**) and baicalein-treated groups in ascending doses (**c**-**e**) showed delayed healing, with a large osteotomy gap in the OVX group, whereas in the baicalein-treated groups, the osteotomy gap was smaller and the callus more compact and denser, primarily at the edges. The red box shows an exemplary bridged callus area on day 26. In the NON-OVX group, bone healing at the advanced stage showed a compact dense callus around the gap (NON-OVX *n* = 9, OVX *n* = 9, C1 *n* = 7, C2 *n* = 7, C3 *n* = 10)

After osteotomy, fluorochromes were applied to visualize bone formation. Using microscopy, the callus area and first osseous bridges were detected (Fig. 5). Osseous bridging was observed in 67% of NON-OVX rats, whereas in the OVX, C1 and C3 groups, the occurrence of the bridging ranged from 40 to 57% (Tab. 1). The highest occurrence of osseous bridging was observed in the C2 group. First osseous bridges of osteotomy gaps were detected between days 20 and 28. The differences between the groups were not significant. In the NON-OVX group, the first bridges could be seen after 20.8 (±6.3) days, whereas in the OVX group, the first bridges occurred after 27.8 (±1.5) days on average. In baicalein-treated groups, bridges appeared on day 24–26 (±4.6) (Tab. 1).

**Fig. 6:**
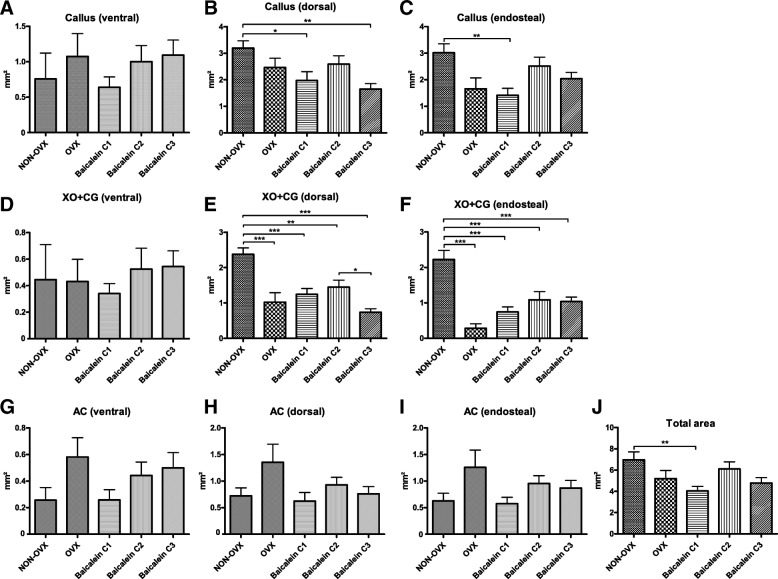
Histological analyses of fluorochrome-labelled sections of tibiae. The callus area was measured in ventral (**a**), dorsal (**b**) and endosteal aspects (**c**). Early stage of healing was measured in ventral (**d**), dorsal (**e**) and endosteal (**f**) aspects by XO + CG labelling and the late stage of healing by AC labelling (**g**-**i**). Finally, the total callus area was calculated (**j**). ***p ≤ 0.001, **p ≤ 0.01, *p ≤ 0.05 (NON-OVX *n* = 11, OVX *n* = 9, C1 *n* = 14, C2 *n* = 12, C3 *n* = 18)

Analysis of the ventral callus area showed a slightly but non-significantly larger area in the baicalein-C3 group compared to the NON-OVX, OVX and C1 groups (Fig. 6a, Additional file 3: Figure S3A, D), while the dorsal area showed an inverse significant effect (Fig. 6b, Additional file 2: Figure S2A, B and Additional file 3: Figure S3B, E). The endosteal callus was smaller in the C1 group than in the NON-OVX-group (Fig. 6c, Additional file 3: Figure S3C, F). In the early healing stages (XO + CG-labelling), OVX led to significantly attenuated healing in the endosteal and dorsal aspects (Fig. 6e, f, Additional file 2: Figure S2A, B, Additional file 3: Figure S3B, C); notably, the highest baicalein dose even worsened this phenomenon (Fig. 6e). In the late bone healing (AC labelling), no statistically relevant differences were measured (Fig. 6g-i), whereas the total area was reduced at the lowest baicalein dose compared to that in the NON-OVX group (Fig. 6j). Together, these results indicate that early callus formation is negatively affected by baicalein.

### Microradiography of the tibia

**Fig. 7:**
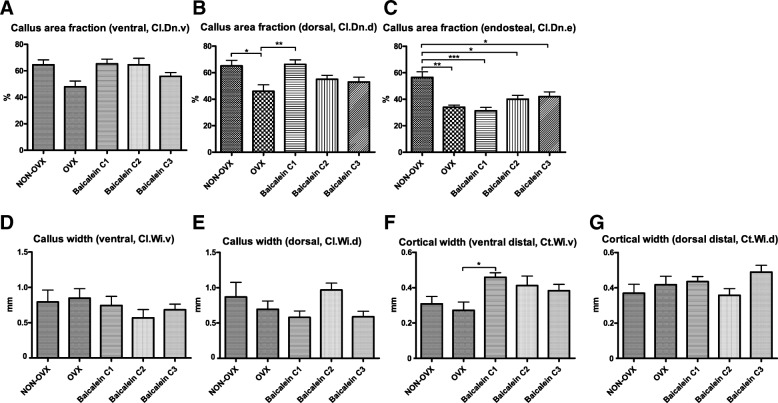
Microradiography of the tibia. Callus area fraction was analysed on ventral (**a**), dorsal (**b**) and endosteal aspects (**c**). Callus width was measured ventrally (**d**) and dorsally (**e**), and cortical width was measured ventral distally (**f**) and dorsal distally (**g**). ***p ≤ 0.001, **p ≤ 0.01, *p ≤ 0.05 (NON-OVX *n* = 12, OVX *n* = 6, C1 *n* = 16, C2 *n* = 14, C3 *n* = 22)

Furthermore, tibial healing was assessed by microradiography to further analyse callus characteristics. The measured callus did not differ in the ventral parameters (Fig. 7a) among the groups. The dorsal callus area fraction, however, was significantly higher in the lowest baicalein dose compared to OVX (Fig. 7b). Endosteal callus area fraction was reduced in all OVX groups (Fig. 7c). Callus width did not differ among groups in the ventral or dorsal part (Fig. 7d, e), but the cortical width showed similar results to the dorsal callus area fraction with increased width in the baicalein C1 group compared to the OVX group (Fig. 7f). The dorsal cortical width (Fig. 7g) and the dorsal and ventral cortical density (data not shown) did not show any significant difference.

### Ashing of the femur

After micro-CT analysis, the femora underwent ashing to assess the organic and anorganic mass as well as mineral components. The wet mass of femora did not differ among the groups before ashing (Additional file 2: Figure S2C).

**Fig. 8:**
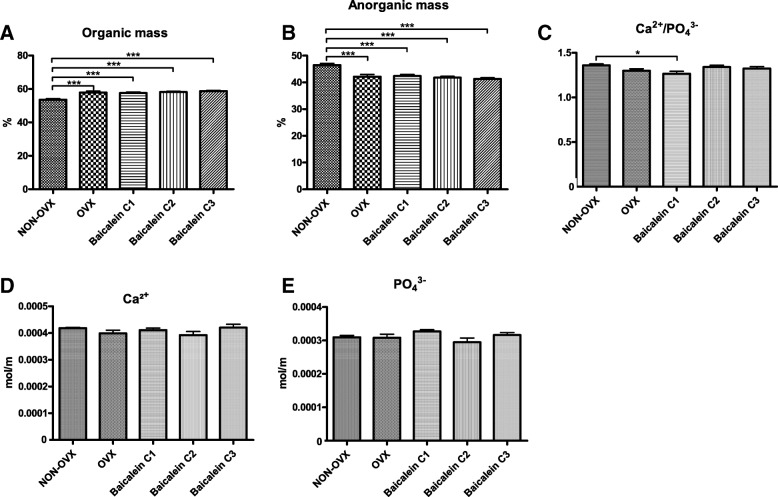
Ashing of the femora. Organic mass (**a**), anorganic mass (**b**), calcium content (**c**), phosphate content (**d**), and calcium/phosphate ratio (**e**). ***p ≤ 0.001, **p ≤ 0.01, *p ≤ 0.05 (NON-OVX *n* = 10, OVX *n* = 9, C1 *n* = 10, C2 *n* = 9, C3 *n* = 10)

Organic mass was significantly higher in all OVX groups (Fig. 8a), and correspondingly, the anorganic mass was lower (Fig. 8b), indicating that anorganic bone mass was significantly reduced by OVX and not altered by baicalein. Calcium and phosphate measurements revealed no significant differences (Fig. 8c, d). The ratio, however, was significantly reduced in the C1 baicalein group (Fig. 8e).

### Gene expression analysis of the tibia at the osteotomy site

**Fig. 9:**
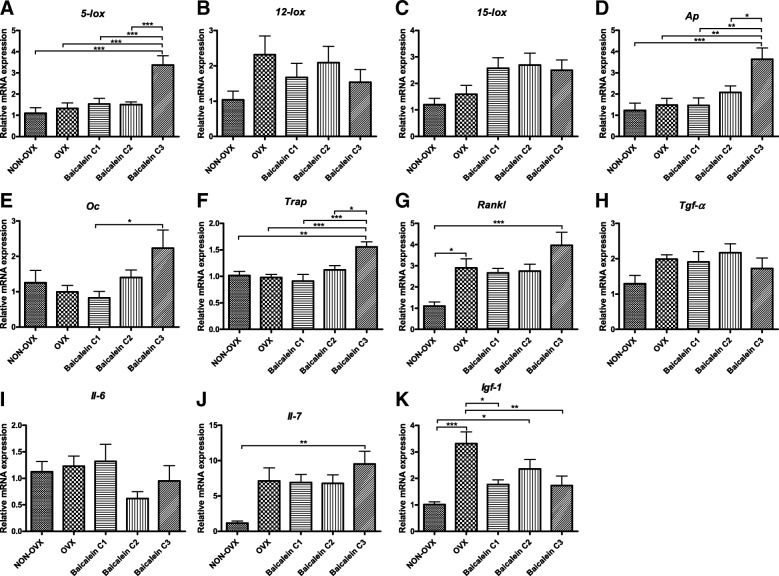
Gene expression analysis at the osteotomy site of the tibia. The expression of arachidonate 5-lipoxygenase (*5-lox*, **a**), 12-lipoxygenase (*12-lox*, **b**) and 15-lipoxygenase (*15-lox*, **c**) were determined to analyse the baicalein effect at the mRNA level. Furthermore, the expression of genes involved in bone metabolism, such as alkaline phosphatase (*Ap*, **d**), osteocalcin (*OC*, **e**), tartrate-resistant acid phosphatase (*Trap*, **f**), receptor activator of nuclear factor kappa B ligand (*Rankl*, **g**), transforming growth factor alpha (*Tgf-α*, **h**), interleukin-6 (*Il-6*, **i**), interleukin-7 (*Il-7*, **j**) and insulin-like growth factor (*Igf-1*, **k**), were analysed. ***p ≤ 0.001, **p ≤ 0.01, *p ≤ 0.05 (NON-OVX *n* = 8, OVX *n* = 9, C1 *n* = 9, C2 *n* = 8, C3 *n* = 8)

Baicalein had no effect on the mRNA level of 12- or 15-lipoxygenase (Fig. 9b-c). In contrast, *5-lox* was significantly increased in the C3 baicalein group (Fig. 9a). Several markers of bone catabolism, such as *Trap* (Fig. 9f), *Rankl* (Fig. 9g) and *Il-7* (Fig. 9j), showed the highest mRNA expression levels after using the highest baicalein concentration. *Ap* (Fig. 9d) and *Oc* as markers of bone formation showed significantly higher values in the C3 group (Fig. 9e). Differences in *Tgf-α* and *Il-6* could not be detected between the groups (Fig. 9h, i). *Igf-1* showed highest values in the OVX group, whereas baicalein treatments C1 and C3 reduced the *Igf-1* expression to the level of that in NON-OVX rats (Fig. 9k).

## Discussion

In the current investigation, we examined the impact of different baicalein doses on bone properties and healing of osteoporotic bones. For this purpose, osteoporosis induced in rats by ovariectomy was successfully verified based on lumbar spine parameters [19].

The exact molecular pathway in which the phytochemical agent baicalein acts in bone is not fully understood. Predominantly and along with 12- and 15-LOX, inhibition of 5-LOX via p38 phosphorylation [12] has been discussed. As a phytochemical agent, baicalein further inhibits cyclooxygenases (COX1 and 2) and thereby the synthesis of prostaglandins and thromboxanes from arachidonic acid [15, 46]. Fracture healing in long-term COX2 deficiency was demonstrated to be impaired [47]. In contrast, reduction of 5-LOX is associated with enhanced fracture healing, including elevated callus chondrogenesis and osteogenesis [11, 48, 49]. Somjen et al. showed that baicalein blocks parathormone (PTH)-induced DNA synthesis unless 12- and 15-lipoxygenase are added [50]. Moreover, baicalein plays a role in inflammatory signalling by inhibiting cytokines such as IL-6 and TNF-α, as demonstrated in vivo by Hu et al. [51]. Although its molecular function remains to be found, baicalein was discovered as an inhibitor of osteoclast differentiation/inductor of osteoclast apoptosis [17] and as a stimulator of osteoblast differentiation in vitro [52]. Its further actions include the suppression of apoptosis in chondrocytes [16]. Li et al. extended our understanding by investigating baicalein’s effect in vivo after detecting an osteogenic effect. The other studies concerning baicalein in bone address aberrant osseous cells, especially osteosarcoma [53, 54]. We showed that baicalein improved cortical bone in lumbar vertebrae, increased the bone formation rate by an enhancing serum alkaline phosphatase and had a favourable effect on skeletal muscle structure [19, 20]. The effects on long bone properties and bone healing have not been discussed so far.

In this study, we first analysed the biomechanical properties of intact femora and osteotomized tibiae. Baicalein altered neither the stiffness nor the maximum force of tibiae at the osteotomy site but impaired stiffness in the femora in highest concentration. This effect confirms the findings of our group about its biomechanical properties in vertebrae [19]. In the tibial micro-CT, neither BV/TV, total BMD nor cortical BMD was significantly reduced after OVX and baicalein treatment, whereas callus BMD was significantly reduced in the OVX group. In contrast to these findings, femoral micro-CT showed reduced BV/TV, total BMD and trabecular BMD in all baicalein-treated groups, which may also be rooted in the OVX effect. Cortical BMD was not altered in femora. This does not match our findings in rat vertebrae, which revealed increased cortical BMD but lowered trabecular connectivity and trabecular BMD in baicalein-treated rats [19]. One possible explanation for why the effect of baicalein is lower in long bones compared to the spine is the bone composition. The vertebra is composed of 25% cortical and 75% trabecular bone, while in long bones the ratio is 50:50 [55]. Further, the bilateral osteotomy performed at the low hind limbs may have some effect on femora.

In the study of Li et al. [18], after a three-month treatment of baicalein using 10 mg/kg BW per day in mice, which corresponded to our C2 baicalein dose, trabecular BMD of femora was significantly raised. We were not able to confirm this effect in the femora examined. One reason could be the shorter period of baicalein application (1 month vs. 3 months) or the s.c. application compared to the intragastric application.

Analysing further the tibia microradiographs, it was shown that in bone healing, dorsal callus area fraction was higher in the lowest baicalein dose compared to OVX, whereas the callus area fraction of the endosteal and ventral callus as well as callus width were impaired in all OVX groups. The statistical significance of dorsal callus area fraction in C1 is hardly proof of a baicalein therapeutic effect and could be a type I error of this observation. The width of the callus did not differ in any aspect (ventral or dorsal), while the ventral cortical width was also improved at the lowest baicalein dose. Once more, baicalein appeared to have an impact on cortical bone [18] but not on the callus properties. There are no comparative studies examining the effect of baicalein on callus formation during bone healing, to our knowledge. A possible explanation of the different site effects observed in tibia could be the different load on the callus and cortical bone due to the plate fixation [56–58]. Though the stable fixation of osteotomy was applied, some micromovements could occur at the dorsal site opposite to the plate [27].

To investigate bone/callus formation in more detail, fluorescence labelling was performed. In histological analyses of osteotomized tibiae, osseous healing was studied. We found that OVX delayed bone healing, whereas baicalein seemed to outweigh this OVX-induced effect partially because the occurrence of the osseous bridges was higher, especially in the C2 group, and the first bridging was seen earlier in baicalein-treated groups compared to the OVX group, but without leading to significant results due to the low number of tibiae analysed. Only the tibiae with a hard osseous callus bridge could be analysed histologically. The treatments were stopped 4 weeks after osteotomy because of the side effects of s.c. application (necrotic lesions at the injection sites [20]). At this point, the bone healing is at the early reparative stage, when osseous callus has bridged and stabilized osteotomized bone [59].

Further analyses of fluorochrome-labelled callus area and callus fraction, in contrast to the bridging data, showed that at the early stage of bone healing, baicalein did not improve OVX-induced impaired fracture healing at the middle or lowest dose (compared to OVX). The highest dose significantly reduced the dorsal callus area fraction in the early stage even in comparison to the other baicalein doses. It was partially compensated at the ventral aspect. On the other hand, in the late healing period, there seemed to be no effect of baicalein compared to OVX, whereas in the OVX group, the delayed early healing was compensated by enhanced dorsal and endosteal callus formation observed at the end of the study.

Fracture healing at the metaphyseal site under stable conditions is characterized by endosteal healing with slight periosteal callus formation [59, 60]. The endosteal callus area was reduced in the C1 group during the whole healing period except the late stage. The early bone-healing phase is characterized by an inflammation cascade, which raises concerns regarding the use of analgesic drugs after fracture [61, 62]. Use of baicalein as a lipoxygenase inhibitor may lead to suppressed bone formation, which we could see at the highest baicalein concentration and in early callus formation [63]. Accordingly, the diminished serum creatine kinase in this cohort also indicates this [20]. At the C3 concentration, a reduced dorsal callus coexisted with the highest mRNA *5-lox* level, indicating a lower 5-lox protein level by a disinhibited feedback mechanism. Baicalein exerts its inhibition on 5-lox at the protein level and not at the mRNA expression level, as verified in vivo by Li et al. [64], which supports our findings. An indirect inhibitory effect on 5-LOX was also demonstrated in another study [12]. The increased expression of bone resorption markers such as *Trap* and *Rankl* as well as *Il-7* may explain the phenomenon of reduced early callus formation. However, the finding that 5-lipoxygenase knockout mice developed a callus rapidly and the bone healed faster [11, 48] contradicts this theory and could indicate that the inhibition of 5-LOX is not the only pathway through which baicalein manipulates bone healing. The inhibition of NF-κB by baicalein can have serious adverse effects through the whole bone-healing process [65]. While mRNA levels of Igf-1 were reduced due to baicalein treatment, the effect on bone healing is inconsistent. The anabolic impact seems to dominate [66], and baicalein can inhibit Igf-1 [67], thus leading to a reduction of callus formation.

Another possible reason for reduced early callus formation could be that when applied in high doses, baicalein leads to downregulation of BCL-2 and an increase in BAX and BIM, leading to apoptosis in fast-growing cells, which has been shown in osteosarcoma cells at high (75 μM), but not low, doses (50 μM) [68]. Baicalein decreases the bone-resorptive activity of osteoclasts and inhibits osteoclast differentiation, subsequently promoting osteoclast apoptosis [17, 52]. Inducing osteoclast apoptosis and cell cycle arrest, baicalein leads to inhibited tumour growth in osteosarcoma cells by inhibiting Wnt/β-catenin pathway [69, 70].

Since in bone healing, chondrocyte-to-osteoblast transdifferentiation and limited apoptosis are required [71], the excessive increase in pro-apoptotic markers such as BAX and BIM and the reduction in anti-apoptotic markers such as BCL-2 could impair bone healing. Another possible mechanism could be the inhibition of parathormone-dependent osteoblast growth, which was shown in vitro for baicalein (1 μM) by Somjen et al. [50].

The beneficial effect on alkaline phosphatase as well as osteocalcin at the mRNA level, which we were able to see in the C3 baicalein cohort, has also been found by Li et al. [18] and could, similarly to the increase in 5-LOX5, through a disinhibited feedback mechanism, indicate a reduced protein level, correlating to the catabolic effect that was morphologically found in early callus formation. Due to this, we were able to detect raised levels of Ap in serum of the rats in our study, published elsewhere [19, 20]. The effects which were seen by the group of Li et al. [17] on femoral bone were not confirmed by our analyses, which can be attributed to longer treatment (5 days before plus 3 months after OVX vs. 4 weeks) or the intragastric method of administration (s.c. in our study). The increase in alkaline phosphatase and osteocalcin along with runt-related transcription factor 2 (RUNX2), as osteoblast differentiation markers, in the present study seems to be promoted by stimulation of the mammalian target of rapamycin complex 1 (mTORC1) signalling pathway [18].

Another possible mechanism that is discussed for the abovementioned effects of baicalein is its radical-scavenging effect, which can have protective effect in oxidative stress [72]. In oxidative stress, antioxidative supplementation was demonstrated to accelerate bone healing since the imbalance between osteoblast/osteoclast activity and excessive apoptosis of osteocytes can be found in bone-resorptive diseases such as osteoporosis [73–75]. One hypothesis for the delayed early callus formation could be that the oxidative stress that prolongs the inflammatory period of fracture healing in the beginning was too high for the baicalein-dose to overcome. An explanation for this could be that antioxidants induce alkaline phosphatase, which in turn promotes matrix protein synthesis in osteoblasts [75]. The raised Ap that we measured in tibia callus demonstrates that this mechanism is one possible explanation for the increased callus area fraction and cortical width.

In summary, three different action mechanisms of baicalein have been discussed: first the antioxidative effect via inhibition of 5-, 12-, and 15-LOX, as well as COX1 and COX2; second, osteoclast apoptosis via impairing the Wnt/β-catenin pathway; and third, the radical-scavenging effect.

To finally connect the observations above with the organic and anorganic bone content, ashing of the femur was performed, which did not reveal differences in the baicalein-treated groups compared to the OVX group. The calcium/phosphate ratio in the baicalein C1 group was lower compared to the NON-OVX group, which can be explained by the OVX effect.

## Conclusions

In summary, baicalein did not alter the total or trabecular BMD compared to OVX-induced reduction in femora. Biomechanical properties were diminished after baicalein C3 treatment in femora. The highest baicalein dose showed reduced callus formation in early, but not late, bone formation, which could be molecularly correlated to the higher expression levels of osteocatabolic genes such as *Trap* and *Rankl*. Thus, administration of baicalein is not advisable during the early stage of healing. To determine the exact effects on bone healing, further studies in which baicalein treatments are started at different stages of healing are needed. Other routes of administration, such as oral or intragastric, should be chosen.

## Limitations

In this study, Baicalein was injected s.c., which caused necrotic lesions [20]. Although these side effects did not affect general healthiness and therefore the study was not interrupted early, different routes of administration (i.e., intragastric as in [18]) are advisable for future studies. We cannot exclude systemic inflammatory effects on bone healing, but – if present – they did not affect overall health (as seen by the analyses of body weight, food intake and general conditions [18]. DMSO itself in a high dose could ameliorate post-ovariectomy osteopenia [76], but all of our groups received the same amounts (even OVX and NON-OVX). Cortical width as it is measured in the sagittal plane (Fig. 7f-g) may be quantified more precisely in axial cross-sections. The biomechanical assessment of tibial callus showed too high variations within the treatment group to detect significant differences (Fig. 2a-b). Therefore, other methods of bone analyses, such as micro-CT and histology, should be used to assess bone healing and provide more reliable data.

## Additional files

Additional file 1: **Figure S1.** Biomechanical test of femur (A) and tibia (C, non-osteotomized tibia). Femur and tibia placed on the aluminium base (A, D). Aluminium bases developed for femur (B) and for tibia (E). Roller stamp (C). (PPTX 3330 kb)

Additional file 2: **Figure S2.** Ratio of endosteal callus area to periosteal callus area (A, B); mass of femora before ashing (C) and mass of uteri (D). No significant differences could be detected in the ratio of endosteal callus area to periosteal callus area in early (A) or late callus formation (B) (NON-OVX *n* = 15, OVX *n* = 7, C1 *n* = 15, C2 *n* = 15, C3 *n* = 22). The wet mass of femora was recorded before ashing, and no differences could be detected between the groups (C), while the mass of uteri was significantly higher in NON-OVX control compared to all other ovariectomized groups (D), as demonstrated elsewhere [20] (NON-OVX *n* = 10, OVX *n* = 9, C1 *n* = 10, C2 *n* = 9, C3 *n* = 10). (PPTX 242 kb)

Additional file 3: **Figure S3.** Callus fraction on ventral (plate osteosynthesis, A, D), dorsal site (opposite, B, E) and endosteal site (C, F). In early callus building, while no differences could be detected ventrally (A), the dorsal fraction was impaired by the highest baicalein concentration (B), and endosteal callus building was not impaired compared to the OVX control group (C). In the late phase, callus building was impaired after baicalein treatment in ventral (D) as well as dorsal (E) and endosteal regions (F). (PPTX 250 kb)
