# Supplementary figures and images for: Effect of the lipoxygenase inhibitor baicalein on bone tissue and bone healing in ovariectomized rats
Source: Nutr Metab (Lond). 2019 Jan 11;16:4. doi: 10.1186/s12986-018-0327-2 (PMC6329162; doi:10.1186/s12986-018-0327-2)

## Slide 1
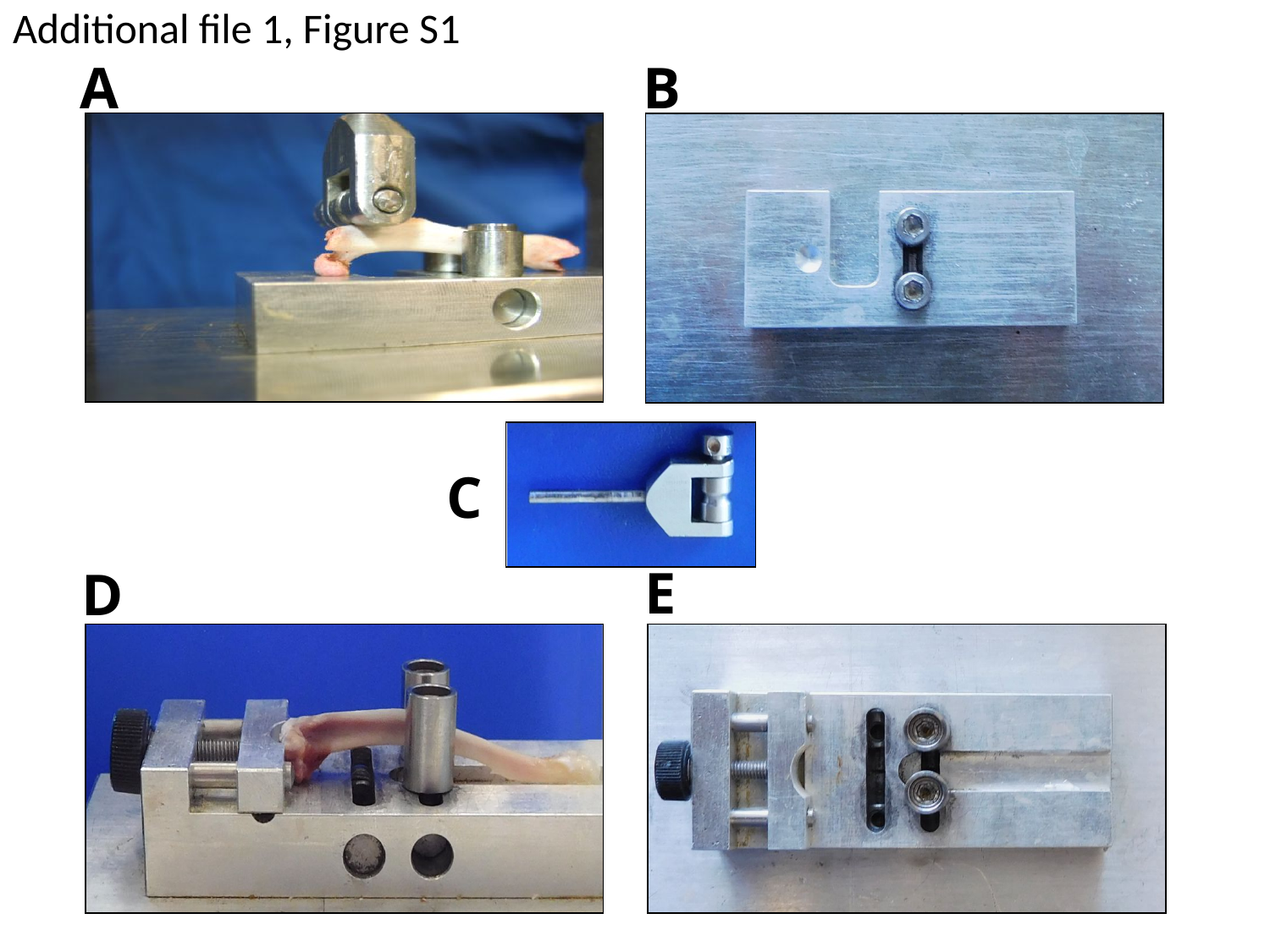

# Additional file 1, Figure S1
A
B
F
E
C
E
D

Supplement: Supplementary file 1 — Figure S1. Biomechanical test of femur (A) and tibia (C, non-osteotomized tibia). Femur and tibia placed on the aluminium base (A, D). Aluminium bases developed for femur (B) and for tibia (E). Roller stamp (C). (PPTX 3330 kb) [file 12986_2018_327_MOESM1_ESM.pptx]

## Slide 1
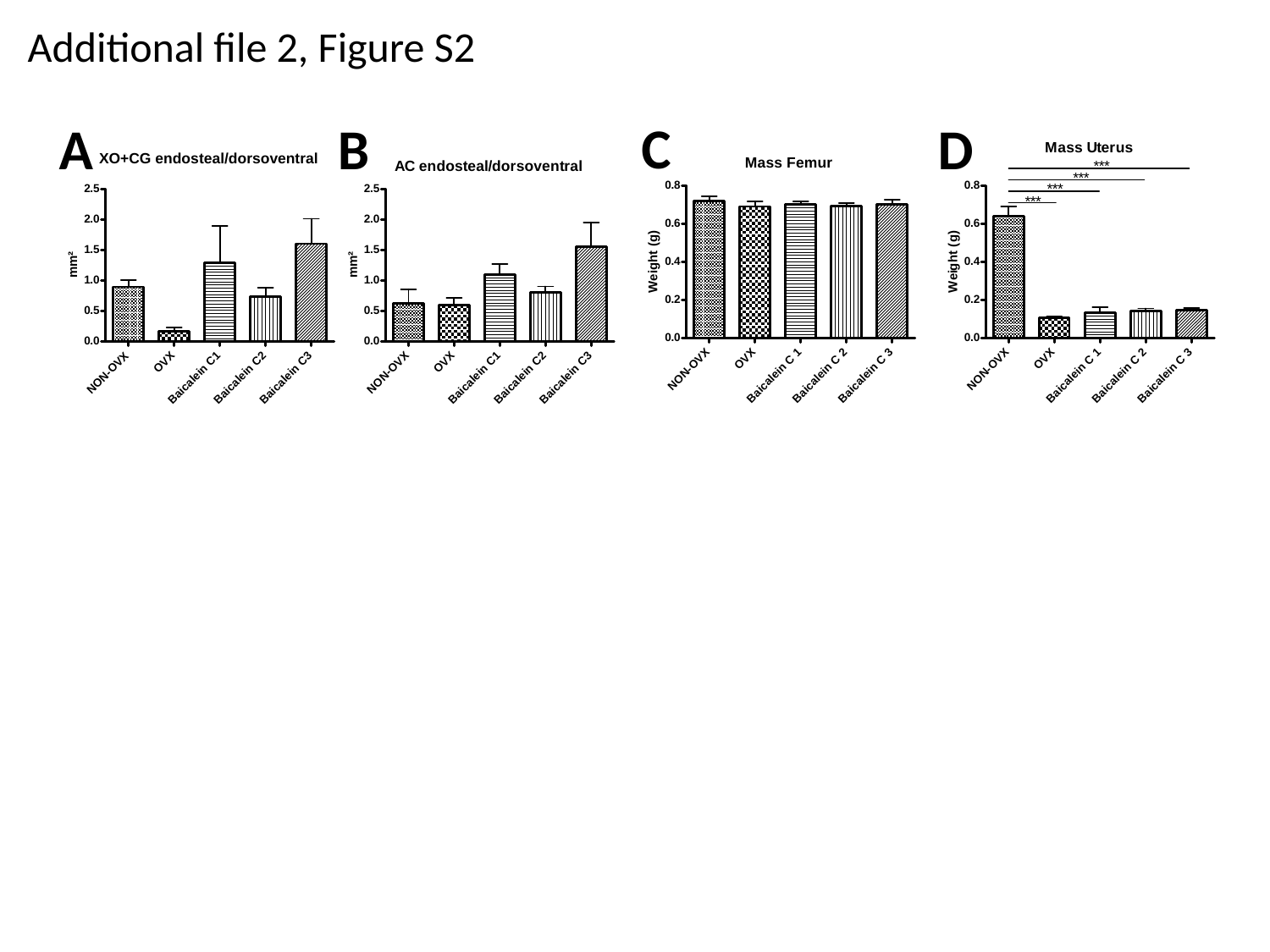

# Additional file 2, Figure S2
C
A
B
D

Supplement: Supplementary file 2 — Figure S2. Ratio of endosteal callus area to periosteal callus area (A, B); mass of femora before ashing (C) and mass of uteri (D). No significant differences could be detected in the ratio of endosteal callus area to periosteal callus area in early (A) or late callus formation (B) (NON-OVX n = 15, OVX n = 7, C1 n = 15, C2 n = 15, C3 n = 22). The wet mass of femora was recorded before ashing, and no differences could be detected between the groups (C), while the mass of uteri was significantly higher in NON-OVX control compared to all other ovariectomized groups (D), as demonstrated elsewhere [20] (NON-OVX n = 10, OVX n = 9, C1 n = 10, C2 n = 9, C3 n = 10). (PPTX 242 kb) [file 12986_2018_327_MOESM2_ESM.pptx]

## Slide 1
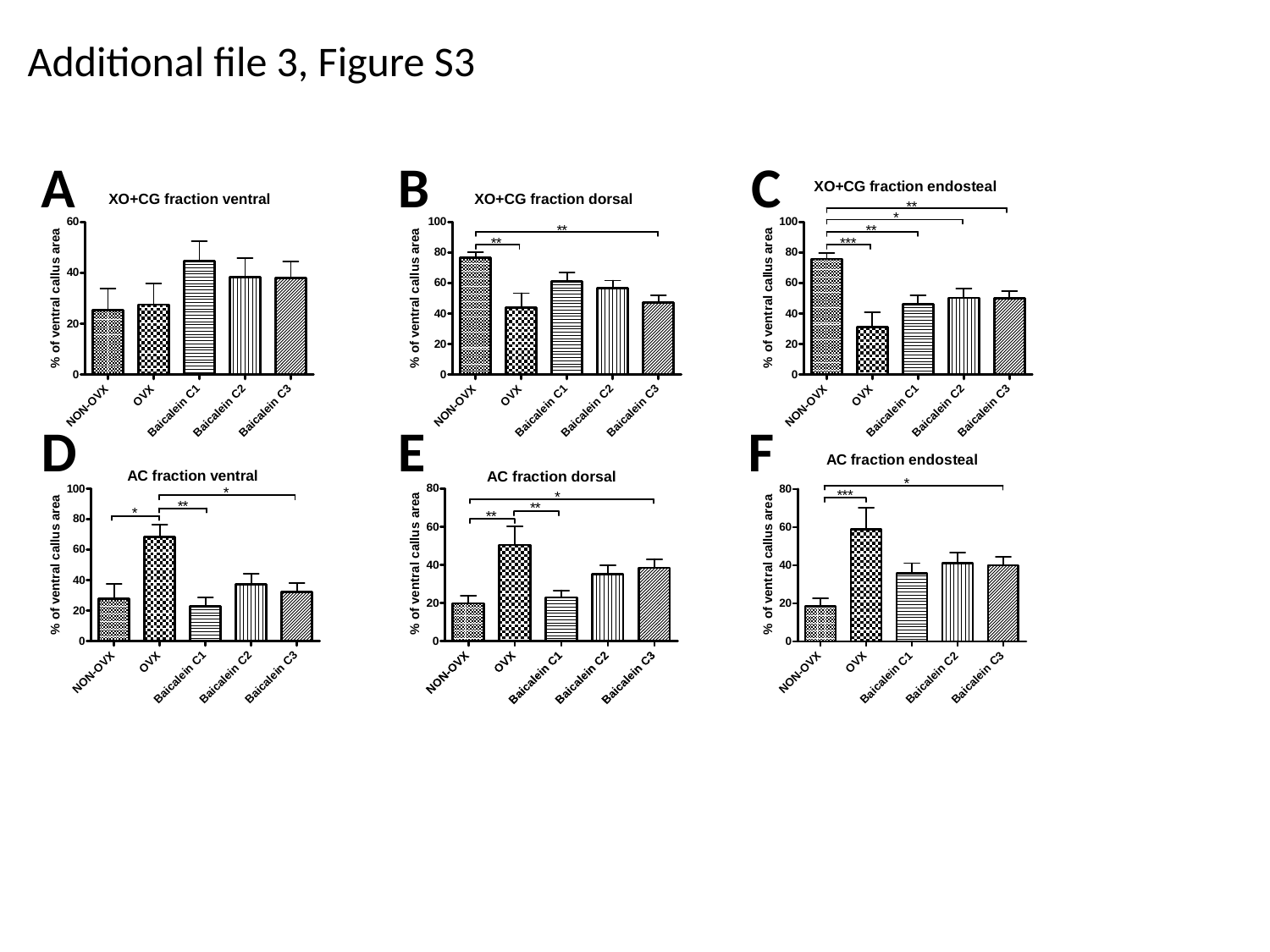

# Additional file 3, Figure S3
A
B
C
F
E
D

Supplement: Supplementary file 3 — Figure S3. Callus fraction on ventral (plate osteosynthesis, A, D), dorsal site (opposite, B, E) and endosteal site (C, F). In early callus building, while no differences could be detected ventrally (A), the dorsal fraction was impaired by the highest baicalein concentration (B), and endosteal callus building was not impaired compared to the OVX control group (C). In the late phase, callus building was impaired after baicalein treatment in ventral (D) as well as dorsal (E) and endosteal regions (F). (PPTX 250 kb) [file 12986_2018_327_MOESM3_ESM.pptx]
